# Peripheral Gating of Nociception Investigated with a Decerebrate, Arterially Perfused Preparation of the Rat

**DOI:** 10.1523/JNEUROSCI.0804-25.2025

**Published:** 2025-08-15

**Authors:** Varinder K. Lall, Pierce Mullen, Stephen W. Milne, Hao Han, Xiaona Du, Nikita Gamper

**Affiliations:** ^1^Faculty of Biological Sciences, University of Leeds, Leeds LS2 9JT, United Kingdom; ^2^ Department of Pharmacology, Hebei Medical University, Shijiazhuang 050011, China; ^3^The Key Laboratory of Neural and Vascular Biology, Ministry of Education, Shijiazhuang 050011, China; ^4^The Key Laboratory of New Drug Pharmacology and Toxicology, Hebei Province, Shijiazhuang 050011, China

**Keywords:** dorsal root ganglion, GABA, nociceptor, pain, peripheral gate, somatosensory system

## Abstract

There is growing evidence that sensory neurons within the dorsal root ganglia (DRGs) are equipped with mechanisms for “gating” nociceptive information before it enters the central nervous system. However, direct in vivo evidence remains limited due to the anatomical inaccessibility of the DRG. Here, we developed a decerebrate, arterially perfused preparation of the rat (of either sex) that allows simultaneous recordings from the C8 spinal nerve (SN) and dorsal root (DR), with full access to the corresponding DRG in the absence of anesthesia. The C8 segment contributes to the median nerve supplying the forepaw, which was used for sensory stimulation. Spikes in DR were recorded and temporally matched to their origin spikes in the SN. Noxious mechanical stimulation to the forepaw increased firing in both the SN and the DR, and application of GABA (200 µM) or the GABA reuptake inhibitor, NO-711 (200 µM), directly into the DRG significantly reduced firing frequency only in the DR without affecting SN activity. Spike sorting revealed that this reduction in the spike rate from SN to DR, a phenomenon we term here as “filtering,” was greater for C-fiber spikes compared with A-fiber spikes. Innocuous stimuli (brush/cotton bud strokes and proprioceptive stimulation) also increased firing in both SN and DR, but GABA application into the DRG failed to reduce DR firing rates. Taken together, our findings lend direct support to the hypothesis of peripheral gating of the nociceptive signaling at the DRG and highlight the therapeutic potential of these peripheral structures.

## Significance Statement

Using an anesthetic-free, decerebrate rat preparation, we demonstrate GABAergic inhibition of nociceptive signal propagation (without effects on innocuous signaling), as these pass the dorsal root ganglion (DRG). These data support the role of neuronal processes at the DRG in gating peripheral pain signals and highlight the DRG's potential as a therapeutic target for pain management.

## Introduction

Spinal nerves (SN) comprise sensory (afferent) axons, which transmit peripheral sensory information to the central nervous system (CNS), and motor (efferent) axons, which convey commands from the CNS to muscles and glands. These nerves emerge from the intervertebral neural foramina between adjacent vertebrae. As the dorsal root (DR) of the primary afferent sensory nerves exits the neural foramina, they form the DR ganglia (DRGs), which house the cell bodies of primary sensory neurons. Each DRG neuron is pseudounipolar: a single axon bifurcates into a peripheral branch, which travels through the SN to innervate peripheral targets (e.g., the forepaw), and a central branch, which projects via the DR into the spinal cord. The soma lies between these branches, connected via a short stem axon to the bifurcation point, the T-junction. While traditionally considered purely afferent, sensory neurons can also generate antidromic (efferent) activity (Sorkin et al., 2018), including neuropeptide release associated with neurogenic inflammation. In trauma or inflammation, DR reflexes can drive efferent signaling from C-fiber afferents, potentially influencing DRG excitability and downstream processing (Yaksh and Di Nardo, 2018).

Traditionally, the spinal cord has been considered the first site of somatosensory processing, with concepts such as the gate control theory of pain dominating explanations of sensory integration (Melzack and Wall, 1965). However, emerging research challenges this view (Moayedi and Davis, 2013; Mendell, 2014; Sundt et al., 2015; Du et al., 2017; Fuller et al., 2023; Hao et al., 2023), suggesting that preliminary “gating” or filtering of peripheral nerve activity may occur earlier, at the DRG T-junction, where spike propagation can fail due to a lowered “safety factor” (Stoney, 1985; Lüscher et al., 1994; Debanne, 2004; Gemes et al., 2013; Sundt et al., 2015; Kent et al., 2018; Hao et al., 2023). Importantly, this failure is dynamically regulated by intrinsic mechanisms, including (but not limited to) the intrinsic GABAergic inhibitory system in the DRG (Du et al., 2017; Hao et al., 2023) and in the trigeminal ganglia (Hayasaki et al., 2006). Such mechanisms may control the amount of nociceptive spikes reaching the spinal cord, making the DRG a promising therapeutic target. This idea underpins the analgesic approach of DRG neuromodulation (Nash, 1986; Liem et al., 2013; Esposito et al., 2019; Kretzschmar et al., 2021). Recent data suggest that both the GABAergic filtering (Hao et al., 2023) and neuromodulation via electric field stimulation (Esposito et al., 2019; Chao et al., 2020, 2021) are more effective in C-fibers (majority of which are nociceptive) as compared with myelinated A-type fibers (many of which mediate innocuous mechanical sensations). Thus, targeting the “DRG gate” may reduce pain while sparing other sensory modalities (Chen and Levine, 2001; Orstavik, 2003; Dubin and Patapoutian, 2010; Du et al., 2017 ; Gossrau et al., 2021).

Studying DRG-based modulation is difficult due to limited in vivo access to the DRG and its pseudounipolar structure being hard to replicate in vitro. Prior in vivo recording approaches successfully evaluated T-junctional failure rates by either electrical stimulation of the SN while measuring DR spikes (Chao et al., 2020; Hao et al., 2023) or by simultaneous measurement of SN and DR activity during peripheral sensory stimulation (Hao et al., 2023). However, these were performed under deep anesthesia, and many anesthetics act directly on GABA_A_ channels (e.g., pentobarbital is a barbiturate that enhances GABAergic inhibition), while commonly used agents like propofol activate or potentiate GABA_A_ channels (Trapani et al., 2000; Matta et al., 2008). These drawbacks complicate understanding of the DRG filtering in an intact nervous system. Thus, we developed the decerebrate, arterially perfused preparation (DAPP), modified from the working heart–brainstem preparation (WHBP; Paton, 1996). DAPP enabled simultaneous recordings from the SN and DR in an anesthetic-free state. These recordings revealed a dynamic GABAergic modulation of nociceptive signals at the DRG, supporting the concept of a peripheral gate within the somatosensory system and its potential as a therapeutic target for pain management.

## Materials and Methods

All animal experiments were approved by the University of Leeds Animal Welfare Ethical Review Body and performed under UK Home Office License P40AD29D7 and in accordance with the regulations of the UK Animals (Scientific Procedures) Act of 1986.

### The DAPP of the rat

Wistar rats of either sex (18–24 d old, University of Leeds in-house animal facility) were deeply anesthetized with isoflurane (Isofane, Henry Schein, 988-3245) until loss of withdrawal reflexes to a firm pinch of the hindpaw using blunt forceps. The arterially perfused preparation was modified from the WHBP (Paton, 1996; Lall et al., 2017). Briefly, after submerging the head and thorax in ice-cold artificial CSF (aCSF) bubbled with a 95% O_2_–5% CO_2_ gas mixture, a decerebration was performed at the precollicular level. Modified aCSF contained the following (in mM): 125 NaCl; 24 NaHCO_3_; 5 KCl; 2.5 CaCl_2_; 1.25 MgSO_4_; 1.25 KH_2_PO4; and 10 d-glucose. In addition, a high molecular weight (70 kDa) oncotic agent 1.25%, Ficoll (type 70; Sigma-Aldrich) was added to the perfusate. The forebrain was removed by aspiration. As the preparation was, at this point, decerebrate, the ongoing use of anesthesia was discontinued. The abdominal cavity was accessed via a midline incision, and the diaphragm, lungs, and surrounding organs were removed. The descending aorta was prepared for cannulation, the thymus was removed, and a ligature was tied between the atria and ventricles to prevent ECG contamination of the electrical signal. Both the ventricles and the atria were removed taking care not to damage the aortic valve. The skin was incised on the dorsal aspect to allow visibility for a laminectomy to be performed. After transfer of the preparation to a recording chamber, the descending aorta was cannulated and perfused with aCSF solution that was heated to 31° (Paton, 1996; Potts et al., 2000), filtered, and passed through two bubble traps using a peristaltic roller pump (Watson Marlow, 520 Du). Perfusion flow rates were between 24 and 30 ml/min, adjusted as appropriate for each preparation based on respiratory rhythm. Rhythmic contractions of respiratory muscles returned within a few minutes after the onset of reperfusion. These were subsequently blocked using the neuromuscular blocker vecuronium bromide (0.04 mg/ml; Norcuron, Organon) to avoid movement artifacts contaminating the nerve recordings. Constant perfusion pressure during DAPP experiments allowed the brainstem to remain adequately oxygenated. A laminectomy was performed along the length of the spinal cord and the area around the C8 DRG was cleared to expose both the SN and DR. These steps took between 10 and 15 min to complete.

### Nerve recordings

**Figure 1.**
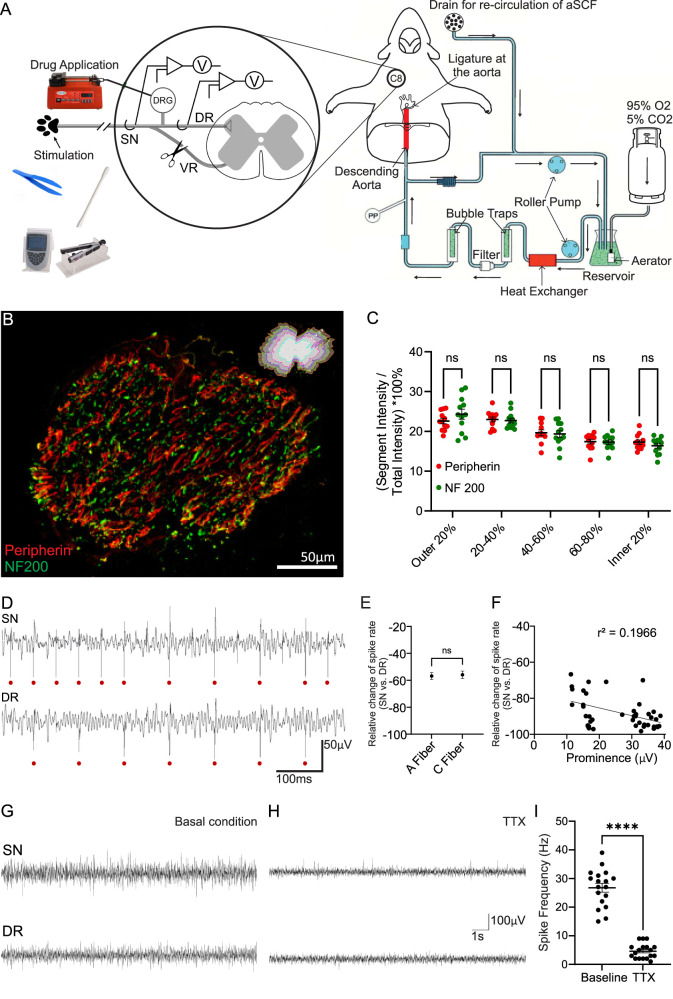
The arterially perfused preparation demonstrates tonic activity at the DRG. ***A***, Cartoon of the DAPP. The descending aorta is cannulated, and the aCSF solution is perfused after being heated, oxygenated, filtered, and passed through bubble traps to ensure continual perfusion of the brainstem and associated circuitry. A ligature was tied at the heart, and the heart was removed, preventing ECG contamination of the recorded signal. The zoomed-in section (left) is a schematic of the surgical exposure of the C8 SN, DRG, and the DR. The forepaw of the rat was stimulated using noxious and innocuous stimuli, while hook electrodes were used to record from the C8 SN and DR. The ventral root (VR) was cut (to avoid motor, efferent information contamination), and drugs such as GABA (200 µM) or NO-711 (200 µM) were injected via sharp electrode directly into the DRG. aCSF flowed out of the cut vessels, drained and recirculated by the roller pump. Adapted from Potts et al. (2000). ***B***, Immunohistochemical staining of the DR nerve cross section revealed no differences in staining patterns of peripherin (red) and NF200 (green), labeling C- and A-fibers, respectively (*p* < 0.99; one-way ANOVA; *n* = 12). ***C***, Quantification of data shown in ***B***. A cross section of the DR nerve was systematically divided into five concentric segments, each representing 20% of the total radial thickness. The outermost 20% constituted the first segment, followed by successive 20% decrements toward the center. Immunohistochemical staining intensity was compared across all five segments; ns denotes no significant difference [*p* = 0.5024 (outer 20%); *p *≥ 0.9999 (20–40%); *p *≥ 0.9999 (40–60%); *p *≥ 0.9999 (60–80%); *p *≥ 0.9999 (inner 20%); 2-way ANOVA; *n* = 12]. ***D***, Extracellular spikes from the SN and DR were identified and counted using the in-house, custom-built software. Red dots denote identified spikes. ***E***, In control recordings (i.e., in the absence of forepaw stimulation and without drug application to the DRG), the relative change in the spike rate from the SN to the DR was calculated for spikes identified as originating from A- and C-fibers using the following formula: % Δ Spikes = (Spikes^SN^ − Spikes^DR^)/Spikes^SN^ × 100; ns denotes no significant difference (*p* = 0.8734; unpaired *t* test; *n* = 26 animals). ***F***, Correlation analysis of relative change in the spike rate from the SN to the DR with spike prominence (*r*^2^ = 0.1966; *Y* = −0.4595 * *X* − 15.09; *p* = 0.0013; simple linear regression; *n* = 15). ***G***,***H***, Effect of TTX on tonic spike activity. ***G***, Example trace of SN and DR activity in control conditions. ***H***, Example traces from the same nerves after perfusion with 1 µM TTX via the descending aorta. ***I***, Quantification of data shown in ***G*** and ***H***. (*n* = 18; *** denotes *p* < 0.0001; paired *t* test). DR, dorsal root; SN, spinal nerve; TTX, tetrodotoxin.

Silver hook electrodes were fashioned in-house and used to record from the DR and SN of the C8 DRG (Fig. 1*A*). The C8 SN contributes to the median nerve innervating the rat forepaw. In all experiments, the ventral root was cut before recordings commenced to eliminate ventral feedback contamination. Nerve signals were recorded using a silver hook electrode attached to a head stage (NL100; Digitimer) and fed into a Neurolog amplifier (1–2K amplification; NL104; Digitimer). Signals were passed through a Humbug (Quest Scientific Technologies) to filter out mains noise at 50/60 Hz. Recordings were sampled at 30 kHz and bandpass filtered between 100 and 3 kHz. All recordings were digitized using an interface [CED 1401; Cambridge Electronic Design (CED)] to be analyzed offline using the Spike2 software (CED) and a custom-written Python script (details below).

### Application of drugs

Solutions of GABA (200 µM, Sigma-Aldrich A2129), NO-711 (200 µM, Abcam, ab120364), and tetrodotoxin (TTX) citrate (1 µM, Bio-Techne, Tocris Bioscience, #1069) were prepared, and aliquots were frozen at −20°C. Prior to each experiment, aliquots were thawed and perfused directly to the DRG using a precalibrated syringe pump [AL-1000, World Precision Instruments (WPI)]. The syringe pump was attached to a sharp glass capillary, mounted on a micromanipulator, which was used to pierce the DRG using the method described earlier (Fischer et al., 2011). During injection, the capillary (∼60 µm tip diameter) was oriented at a 30° angle and advanced until a dimple was observed on the DRG capsule. The capillary was then advanced in small increments until the DRG was pierced. Once penetrated, the capillary was retracted slightly to minimize the compression of the DRG. The capillary was left in place for 5 min until a seal was formed around the tip. To avoid electrical interference and to minimize the leakage of the injected solution, a low toxicity silicone adhesive (Kwik-Sil, WPI) was applied around the DRG, sharp capillary, and both hook electrodes. This electrically isolated the nerve from the bath, allowing electrical signals to be recorded from the nerve while it was submerged in the perfusate solution. Recordings were made for a maximum of 3 h postsurgery.

### Peripheral sensory stimulation

Noxious stimulation was elicited to the forepaw using a portable Randall–Selitto Paw Pressure Meter (WPI, 800G, II-2500). Force was measured in grams and digitized using the CED 1401 to allow correlation with identified spike units. C-fiber could be classified as nociceptive based on their high-mechanical thresholds and sustained firing to the static phase of a ramp and hold force stimulus. Innocuous mechanical stimulation was elicited to the forepaw by either brushing the dorsal surface of the forepaw using a cotton swab for 10 s (here named “soft tactile”) or by brushing the end of a plastic tweezer to the dorsal surface of the forepaw (here named “firm tactile”). Proprioceptive stimulation was elicited via movement of the elbow vertically up and down for 10 s (without noxious stimulation, no force applied to the paw).

### Analysis and statistical tests

Stable periods of baseline DR and SN discharge were obtained. During interventions, five trials per preparation were conducted. During each intervention, activity was analyzed immediately before the stimulus (termed “control”), for a period of 10 or 30 s (as stated) during the stimulus, and immediately after the stimulus (termed “recover”). These data were then expressed as firing frequency (Hz) or as a percentage change from baseline values due to interpreparation variability (as stated). Unless otherwise stated, all statistical analyses were performed using the Excel (Microsoft) or Prism (GraphPad) software. A two-tailed Student’s *t* test was used for single comparisons between two groups (paired or unpaired), and one-way or two-way ANOVA, followed by Tukey’s test, was used for multiple comparisons. Data are represented as mean ± standard error of the mean. Symbols *, **, ***, **** and NS denote statistical significance corresponding to the following *p* values: <0.05, <0.01, <0.001, <0.0001, and no statistical significance, respectively.

### Spike sorting

Spike sorting and matching approach is described in detail previously (Hao et al., 2023). Briefly, extracellular recordings of both the SN and DR were imported into Python and high-pass filtered at 60 Hz using a digital Butterworth filter from the Scipy module. Extracellular spike times and waveforms were extracted using a threshold defined as the absolute median deviation multiplied by a factor of 4 from the median of the signal. Extracted spike waveforms were sorted using the WaveClus program (Chaure et al., 2018) in MATLAB to define individual neuronal units underlying the spiking activity in the extracellular signal. This method of spike matching proved 80–100% accurate in Poisson-generated spike trains up to 100 Hz, irrespective of the degree of T-junctional filtering here (Hao et al., 2023); the accuracy was inversely proportional to firing frequency. This allowed the SN spikes to be grouped into distinct firing clusters or units that could then be evaluated for sensitivity to different stimuli, e.g., thermal and pressure. Stimulus sensitivity of firing units was quantified by regressing the instantaneous firing rate of each firing unit against stimulus regressors (force). To quantify which types of spikes successfully propagated across the T-junction, we used a method of temporally matching DR spikes to their origin spike in the SN, which was achieved by finding the minimum latency (within a tolerance window of the slowest theoretical fiber conduction velocity of 0.1 m/s) between spikes in the DR and spikes in the SN over the estimated distance between the two recording electrodes (4 mm). Units were divided into presumably “C-type” and presumably “A-type” based on the SN–DR latency (C, <2 m/s; A, >2 m/s). The code for spike sorting analysis is available at GitHub (https://github.com/pnm4sfix/SpikePropagation). DR spikes were paired with the nearest preceding SN spike within the tolerance window, and this defined a “match.” The identity of sorted spikes was then extended to the DR spikes after matching, and the propagation success for each firing unit was quantified as the percentage of SN spikes within that firing unit that had a matching DR spike. Propagation failure was defined when a SN spike could not be matched to a spike in the DR, within the tolerance window.

### Immunohistochemistry

The DR was extracted and fixed in 4% PFA (Thermo Fisher Scientific) for 1 h at 4°C. Following fixation, the nerve was saturated in 30% sucrose prior to encapsulation in Frozen Section Compound (Leica Biosystems). Nerve sections were cut using a Cryostat (Leica Biosystems) at a section thickness of 20 µm and loaded onto Super Frost slides (Thermo Fisher Scientific) and stored at −20°C. Cryostat-sectioned tissue samples were immunostained on the microscope slides. Slides were washed with PBS and incubated with primary antibody solution (PBS + 0.2% Triton X-100), supplemented with primary antibodies raised against Neurofilament 200 (NF200; 1:500 dilution; rabbit, N4142, Merck), or peripherin (1:1,000 dilution; chicken, ab39374, Abcam) overnight at 4°C. Primary antibodies were omitted in control. The slides were washed three times in PBS for a minimum of 10 min per wash and incubated at room temperature for 2 h with secondary antibody solution: PBS containing 10 mg/ml BSA supplemented with Donkey anti-rabbit 488 antibody and goat anti-chicken 555 antibody (both 1:1,000 dilution, Life Technologies). After three washes with PBS, mounting medium with DAPI (ab104139, Abcam) was applied to the slide, coverslips were mounted, and slides were stored at 4°C until imaged. Images were acquired as confocal *z*-stacks using a LSM 880 microscope (Carl Zeiss) with a 20× objective. Maximum intensity projection was performed on each *z*-stack. The following lasers were utilized: 405 nm laser for DAPI, 488 nm laser for visualizing NF200 staining, and 561 nm laser for visualizing the peripherin staining. Image quantification was performed in NIS Elements (Nikon Europe BV). To analyze relative distribution of NF200-positive and peripherin-positive fibers within the nerve, each fiber bundle was outlined to create Region of Interest 1 (ROI1) and the area of ROI1 measured. ROI1 was duplicated and resized to either 80, 60, 40, or 20% area of ROI1 (ROI2–5). The fluorescence intensity of each ROI for NF200 and peripherin was measured using the ROI statistic function and exported to Excel, where the fluorescence distribution was calculated.

## Results

### DAPP of the rat

To directly assess the DRG’s ability to filter spike throughput in anatomically intact nerves, without continuous anesthesia and with preserved central connectivity, we modified the WHBP (Paton, 1996). Anesthesia is only administered at the initial stages of preparation and is discontinued after decerebration. In the updated preparation, we removed the heart to prevent ECG contamination of neural recordings, enabling direct recording of somatosensory electrical activity from peripheral nerves. We termed this modified preparation the DAPP of the rat (depicted in Fig. 1*A*). The preparation allows for laminectomy from the lower thoracic to upper cervical levels, facilitating continuous electrophysiological recordings and/or stimulation at various points along the C8 spinal and median nerve innervating the forepaws. DAPP offers complete access (including optical) to the corresponding C8 DRG and its DR. Importantly, the forepaw is fully accessible for diverse sensory stimuli (Fig. 1*A*).

Before extracellular nerve recordings were conducted, it was important to explore the distribution patterns of the smaller, unmyelinated C-fibers versus larger, myelinated A-fibers in the nerve trunk to ensure that recording electrodes are not biased toward one fiber type over another. Cross sections of the DR were stained with peripherin, a marker for the cell bodies and axons of C-fiber neurons and, with NF200, a marker for the myelinated axons of A-type fibers (Fig. 1*B*,*C*). The outline of each nerve trunk cross-section was drawn and then reduced by 20% intervals to create five concentric sections (see Materials and Methods). The peripherin-positive and NF200-positive fibers were distributed approximately evenly throughout the section, with no obvious pattern. Thus, the average relative fluorescence intensity for peripherin and NF200 was comparable in all five sections analyzed (Fig. 1*B*,*C*).

### Tonic activity is higher in the SN

Recordings from the C8 root of the median nerve, capturing both the SN and the DR, using the DAPP revealed sustained tonic extracellular spike firing. Activity was observed in the SN (before an afferent spike enters the DRG) and in the DR (after an afferent spike leaves the DRG), which was consistent with previous recordings in anesthetized rats (Hao et al., 2023). The extracellular action potentials in the SN and DR were counted, sorted, and matched (see Materials and Methods) to identify possible failure to propagate from the SN to the DR. Indeed, regular missing spikes were identified (Fig. 1*D*). Bulk analysis of average firing frequencies in the DR versus SN revealed that firing rates in the DR (10.7 ± 1.2 Hz) were consistently lower than in the SN (25.4 ± 3 Hz; paired *t* test; *p* = 0.007; *n* = 26 animals and 119 time points analyzed; Fig. S1*A*,*B*). The relative change in the spike rate from the SN to the DR was calculated using the following formula: % ΔSpikes = (Spikes^SN ^− Spikes^DR^) / Spikes^SN ^× 100. The mismatch between tonic firing rates in SN and DR segments affected both A- and C-fibers, as identified by spike sorting: the firing frequency in the DR was reduced by 56.8 ± 3% of that in the SN for A-fibers and by 56 ± 3% for C-fibers at the baseline (unpaired *t* test; *p* = 0.87; *n* = 26 animals; Fig. 1*E*). The average drop in the firing frequency from SN to the DR was 48.4 ± 4.1%, which was consistent with previous observations (Hao et al., 2023). Spikes with the greatest prominence were observed to have a greater relative change in the spike rate from SN to DR in control conditions (simple linear regression; *r*^2^ = 0.1966; *Y* = −0.4595 * *X* − 15.09; *p* = 0.0013; *n* = 15; Fig. 1*F*). The tonic activity was almost completely abolished by the voltage-gated Na^+^ channel blocker, TTX (1 µM; Fig. 1*G*,*I*), delivered via the arterial perfusion system to ensure consistent exposure throughout the preparation, including the DRG and associated nerves. These results indicate that at control conditions (the absence of external sensory stimulation), there is a significant level of ongoing spontaneous activity at the peripheral nerve; about half of it fails to propagate beyond the DRG. It must be noted that tonic activity recorded in DAPP may not fully represent the true basal state.

### GABA_A_ channel activation has little effect on the tonic activity of the nerve

**Figure 2.**
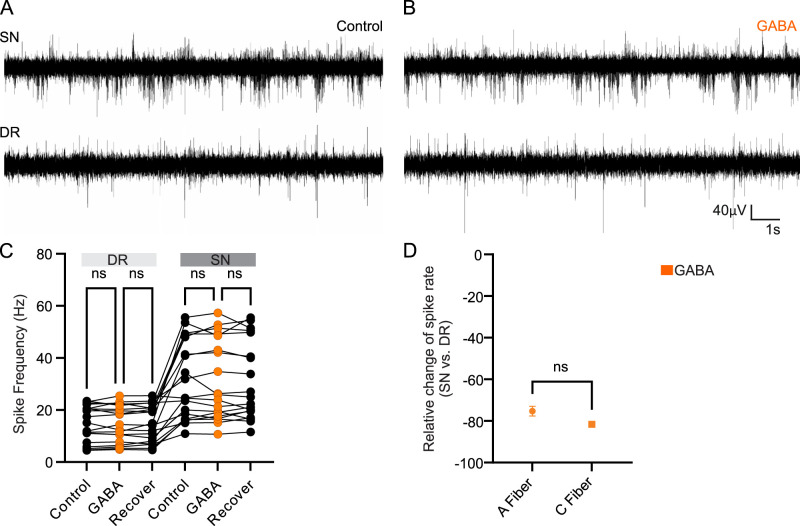
GABA has no effects on baseline activity in either the DR or the SN. ***A***,***B***, Example traces of SN and DR during control (***A***) and (***B***) GABA (200 µM) infused directly into the DRG. ***C***, Quantification of spike frequency from the recordings as in ***A*** and ***B***; DR, *p* = 0.9960; SN, *p* > 0.9999; one-way ANOVA; ns denotes no significant difference; *n* = 19 animals and 51 stimulations. ***D***, Quantification of the relative change in the spike rate from SN to DR; ns denotes no significant difference (*p* = 0.1330, unpaired *t* test). GABA, γ-aminobutyric acid.

We next tested the effect of GABA on the tonic activity and the spike propagation at the DRG. GABA (200 µM, 20 µl/min) was infused directly into the DRG via a sharp glass capillary for a period of 30 s. A “control” period was taken for 30 s prior to GABA application and a 30 s “recover” period was taken 5 s after GABA infusion had stopped. Direct injection of GABA during tonic/control activity (no sensory or other stimulations) did not alter firing frequencies in either the DR or the SN (DR, 14.7 ± 1.6 Hz control versus 14.8 ± 1.6 Hz in GABA; *p* = 0.996; SN, 31.2 ± 3.4 Hz control vs 31.2 ± 3.5 Hz in GABA; *p* > 0.999; one-way ANOVA; *n* = 19 animals, 51 stimulations analyzed; Fig. 2*A*–*C*). Thus, GABA injected directly into the DRG has no effects on spike propagation through the DRG in the absence of peripheral stimulation (during tonic/control conditions). To confirm that the infusion of a liquid into the DRG does not itself alter firing frequencies in either the DR or the SN, we directly injected the vehicle (aCSF) into the DRG using the exact same protocol as for GABA injection. As expected, vehicle infusion into the DRG had no effects on spike frequency in either the DR or the SN, and there were no predictors of filtering (Fig. S1*C*,*D*).

### GABA increases filtering of noxious but not innocuous mechanical stimulation of the rat forepaw

In the next series of experiments, we tested how DRG-applied GABA modulates the neural responses evoked by noxious and innocuous stimulation of the front paw. Noxious mechanical stimulation was elicited by a portable Randall–Selitto Paw Pressure Meter (WPI, 800G, 11-2500). To determine the force of pressure required to elicit a noxious withdrawal response, we first conducted experiments in awake animals. A noxious response was noted as a vocalization, withdrawal, or a freezing response. On average, a force of 125 ± 10 g was required to produce a noxious response in awake animals (*n* = 35 trials; nine animals; Fig. S2), which is in agreement with previous studies (Santos-Nogueira et al., 2012).

**Figure 3.**
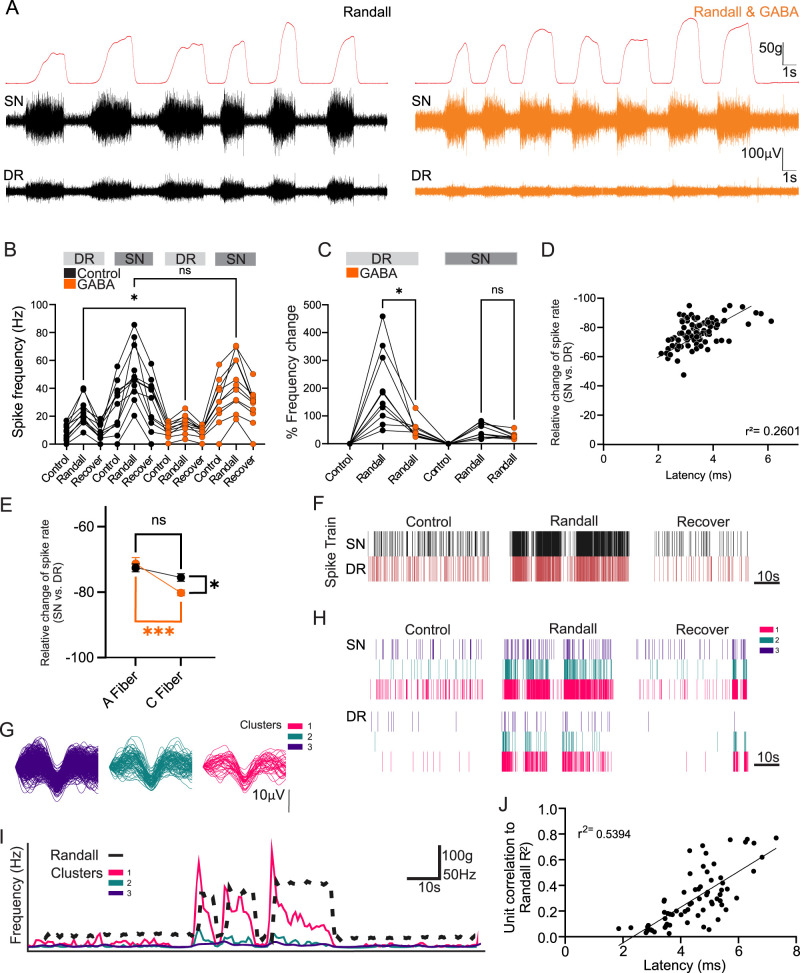
GABA application at the DRG increases spike filtering, particularly affecting the C-fibers. ***A***, Left, Recordings of the nerve activity induced by noxious Randall stimulation at the forepaw. Top trace (in red) shows mechanical stimulation applied by the Randall in grams. Middle trace is the SN, and bottom trace is the DR. Right, Randall stimulation at the forepaw with GABA (200 µM, 20 µl/minute; orange traces) injected directly into the DRG. ***B***, Quantification of spike frequency in the DR and SN from data shown in ***A*** and ***B*** (* denotes *p* < 0.05; DR, *p* = 0.0330; ns denotes no significant difference; SN, *p* = 0.6622; one-way ANOVA; *n* = 11 animals). ***C***, Quantification of the percentage change in spike activity (SN to DR) during control, Randall stimulation and Randall stimulation in the presence of GABA (* denotes *p* < 0.05; ns denotes no significant difference; one-way ANOVA; *n* = 15). ***D***, The latency between SN and DR spikes was calculated and correlated with the probability of SN to DR loss. Each spike in the DR was paired with a spike in the SN based on the minimum latency within a short time window (defined by an estimation of the slowest conduction velocity of C-fibers). This avoided matching temporally uncorrelated spikes during gaps in spike activity. Using this method, the minimum latency defined the SN origin of a DR spike. Spike sorting extracellular SN waveforms using the WaveClus implementation of superparametric clustering allowed us to isolate distinct spike clusters (units) and match these between SN and DR recordings. Units were divided into “C-type” and “A-type” based on the SN–DR latency and propagation failure rate analyzed (*r*^2^ = 0.2601; *p* < 0.0001; *Y* = −6.104 * *X* − 55.65). ***E***, Extracellular spike waveforms extracted from a SN recording and clustered using WaveClus were compared for the failure rate (between “A”- and “C”-fibers) upon Randall stimulation with and without GABA; * denotes *p* < 0.05; *** denotes *p* < 0.0001; ns denotes no significant difference. (A-fiber Randall vs A-fiber Randall in GABA *p* = 0.8961; C-fiber Randall vs C-fiber Randall in GABA *p* = 0.0366; A-fiber Randall vs C-fiber Randall *p* = 0.3252; A-fiber Randall in GABA vs C-fiber Randall in GABA *p *≤ 0.0001; one-way ANOVA). ***F***, Instantaneous firing frequency of all sorted spike units in the DR (red) and SN (black) from a single representative recording. ***G***, Individual waveforms of spike sorted units (clusters) in the SN identified using WaveClus, from the representative example in ***F***. ***H***, Raster plot for each clustered waveform (denoted as Unit) under control, Randall, and recover matching the DR spikes with those in the SN, from the representative example in ***F***. ***I***, Firing frequency of the three clusters identified in ***G*** (in color), with Randall trace superimposed (in black). ***J***, For each mechanosensitive cluster identified, the correlation to the Randall trace (*r*^2^ value) was calculated and correlated to latency (*p* < 0.001; *r*^2^ = 0.5394; *Y* = 3.884 * *X* + 3.339). DR, dorsal root; SN, spinal nerve. GABA, γ-aminobutyric acid.

Next, using DAPP and dual DR–SN recording, the dorsal and plantar surfaces of the central paw (not the digits, as in Fig. S2) were squeezed using the paw pressure meter which recorded the grams of force/pressure applied (Fig. 3*A*, top traces). A minimum force of 125 g was applied in each noxious stimulation in the DAPP. Extracellular spikes were identified and counted in the DR and SN as described in “Materials and Methods.” Upon noxious mechanical stimulation, the average firing frequency of the DR increased 158% from control levels of 8.9 ± 1.7 Hz to 22.9 ± 3 Hz. When GABA (200 µM) was continually infused into the DRG, the increase in DR firing frequency upon noxious Randall stimulation was significantly attenuated to an increase of 49% or 14.9 ± 2 Hz (one-way ANOVA; *p* = 0.033; *n* = 50 stimulations; 11 animals; Fig. 3*A*–*C*). It was noted that those animals with the highest tonic DR firing rates responded to GABA with the most attenuated firing frequency. The average firing frequency of the SN also increased upon Randall stimulation, from 26.5 ± 5.3 Hz to 50.7 ± 6 Hz; however, this increase remained unchanged with GABA at 44.6 ± 6 Hz (one-way ANOVA; *p* = 0.662; *n* = 50 stimulations; 11 animals; Fig. 3*A*–*C*). Thus, DRG-applied GABA specifically reduced nociceptive response to noxious mechanical stimulation in the DR but not the SN. Here, we refer to this phenomenon as GABA-dependent filtering of spikes at the DRG.

Extracellular spikes identified and counted in the DR were matched back to an origin spike in the SN, which allowed identification of distinct spike clusters or units. The relative change of the spike rate (SN vs DR) was greatest in those spike clusters with the longest latencies (*R*^2^ = 0.2601; *Y* = −6.104 * *X* − 55.65; *p* < 0.0001; Fig. 3*D*). When characterizing the spike units based on conduction velocity, we saw that the relative change in the spike rate from SN to DR during noxious Randall stimulation was greater for slower spikes (identified as C-type), as compared with the faster spikes (identified as A-type; A-fiber Randall vs A-fiber Randall in GABA *p* = 0.8961; C-fiber Randall vs C-fiber Randall in GABA *p* = 0.0366; A-fiber Randall vs C-fiber Randall *p* = 0.3252; A-fiber Randall in GABA vs C-fiber Randall in GABA *p* = <0.0001; one-way ANOVA; Fig. 3*E*), which was consistent with previous observation (Hao et al., 2023). A representative example of sorted spikes is shown in Figure 3*F*–*H*. In this example, spike frequency was calculated, and a raster plot of spikes was produced (Fig. 3*F*). From this, three clusters or units were identified based on spike waveforms using the WaveClus program (Fig. 3*G*), and a raster plot for each clustered waveform was produced from this (Fig. 3*H*). The latency of each identified cluster was calculated and used for fiber-type assignment. To quantify the individual units as being mechanosensitive, we correlated the Randall traces (force applied) with the traces generated from individual clusters. Plotting the *r*^2^ values and latency demonstrated that units or clusters with the longest latencies, likely to be originating from the C-fibers, were the most correlated to the Randall trace and, therefore, responded to the increase in mechanical force applied using the Randall meter more so than other units (*r*^2 ^= 0.5394; *Y* = 3.884 * *X* + 3.339; *p* < 0.0001; *n* = 67 clusters; Fig. 3*I*,*J*).

Of note, noxious stimulation reduced the mismatch of firing frequencies between SN and DR: in control conditions, firing in DR was 44 ± 4.5% of that in SN, while upon noxious Randal stimulation, the firing in DR has risen to 55 ± 4.6% of the in SN (paired *t* test; *p* = 0.000594; Fig. S1*A*,*B*). Taken together, these data reveal that the firing rates of the SN are consistently higher than the DR (suggesting a degree of tonic filtering), and mechanical noxious stimulation increases firing rates in both the SN and the DR, during which, the relative change in the spike rate from SN to DR decreases. This implies the degree of tonic filtering is lower during noxious stimulation compared with the baseline. Finally, DRG-applied GABA preferentially filters those units identified as originating from the C-fibers.

**Figure 4.**
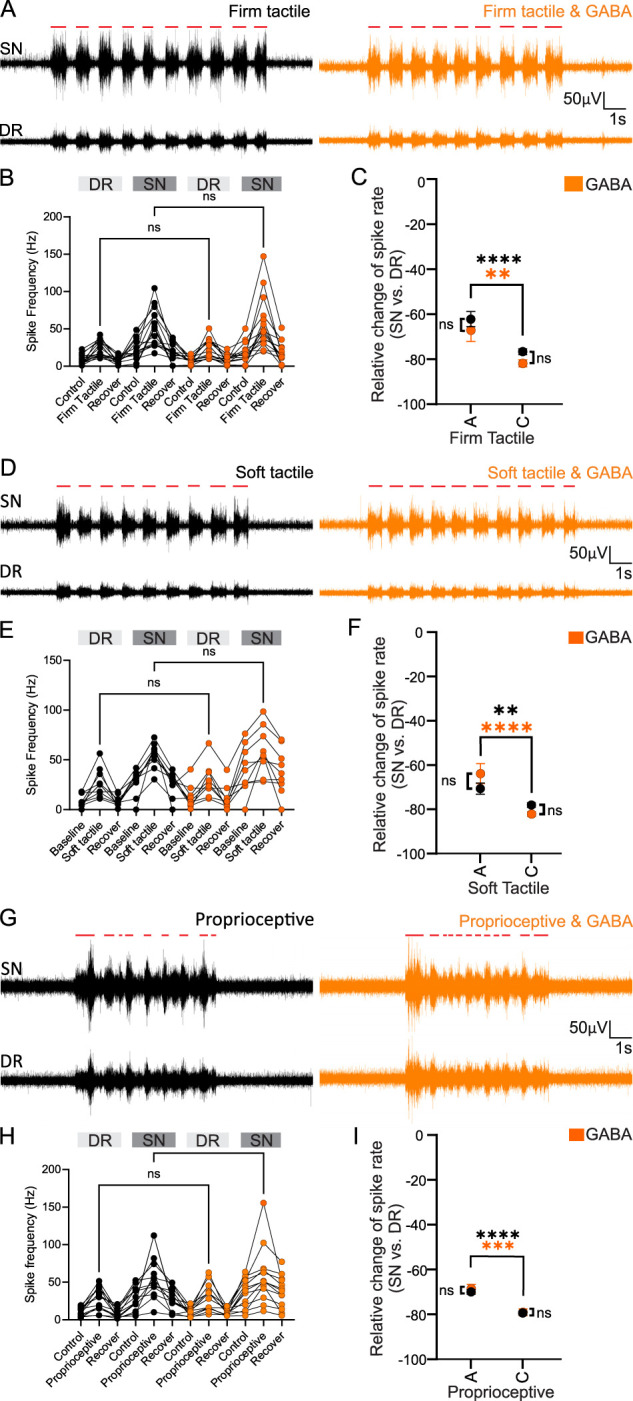
GABA application at the DRG has no effects on activity induced by innocuous stimuli. ***A***, Example traces of nerve activity induced by firm innocuous tactile stimuli (red lines) applied to the forepaw of the rat in the SN (top traces) and DR (bottom traces); black traces, control; orange traces, GABA (200 µl) applied to the DRG. ***B***, Quantification of firing frequencies from the data exemplified in ***A***; ns denotes no significant difference (DR, *p* > 0.9999; SN, *p* > 0.9999; one-way ANOVA; *n* = 15 animals). ***C***, The relative change in the spike rate from SN to DR in A- or C-fibers upon firm tactile stimulation with or without the presence of GABA; **** and ** denote *p* < 0.0001 and *p* < 0.01, respectively; black symbols denote comparison of stimulation under control conditions; orange symbols denote stimulation under GABA infusion into the DRG; ns denotes no significant difference (A-fiber, *p* = 0.6919; C-fiber, *p* = 0.1566; *n* = 15 animals). ***D***, Example traces of nerve activity induced by soft tactile stimulation (red lines) applied to the forepaw of the rat in the SN (top traces) and DR (bottom traces); color code as in ***A***. ***E***, Quantification of firing frequencies from the data exemplified in ***D***; ns denotes no significant difference (DR, > 0.9999; SN, *p* > 0.9999; one-way ANOVA; *n* = 9 animals). ***F***, The relative change in the spike rate from SN to DR in A- or C-fibers upon soft tactile stimulation with or without the presence of GABA; **** and ** denote *p* < 0.0001 and *p* < 0.01, respectively; black symbols denote comparison of stimulation under control conditions, orange symbols denote stimulation under GABA infusion into the DRG; ns denotes no significant difference (A-fiber, *p* = 0.2650; C-fiber, *p* = 0.0694; *n* = 9 animals). ***G***, Example traces of nerve activity induced by proprioceptive stimulation (red lines) applied to the forepaw of the rat in the SN (top traces) and DR (bottom traces) elicited by moving the forearm vertically up and down without noxious stimulation; color code as in ***A***. ***H***, Quantification of firing frequencies from the data exemplified in ***G***; ns denotes no significant difference (DR, *p* > 0.9999; SN, *p* > 0.9999; *n* = 13 animals). ***I***, The relative change in the spike rate from SN to DR in A- or C-fibers upon proprioceptive stimulation with or without the presence of GABA; **** and *** denote *p* < 0.0001 and *p* < 0.001, respectively; black symbols denote comparison of stimulation under control conditions; orange symbols denote stimulation under GABA infusion into the DRG; ns denotes no significant difference (A-fiber, *p* = 0.9371; C-fiber, *p* = 0.9976; *n* = 13 animals). DRG, dorsal root ganglia; DR, dorsal root; SN, spinal nerve.

To examine the effects of GABA on propagation of spikes induced by innocuous stimulation, soft and firm tactile mechanical stimulation was applied to the forepaw. Firm tactile stimulation was applied using the end of plastic tweezers to stroke the dorsal surface of the forepaw, being gentle enough not to elicit a noxious response (no pinching of the paw). Firm, innocuous tactile stimulation elicited a robust increase in spike activity in both the DR (from baseline levels of 8.2 ± 1.7 Hz to 23.6 ± 2.7 Hz) and the SN (from baseline levels of 20 ± 3.7 Hz to 51.4 ± 6.5 Hz; *n* = 15 animals). With firm tactile stimulation in the presence of GABA, both the DR and SN firing frequency remained unchanged (DR, 23.6 ± 3.3 Hz; *p* > 0.999; SN, 56.7 ± 9.2 Hz; *p* > 0.999; *n* = 15 animals; Fig. 4*A*,*B*). Spike sorting revealed that during firm tactile stimulation, there was no difference in the degree of filtering of those fibers identified as originating from either A- or C-fibers, and application of GABA during tactile stimulation did not significantly affect filtering (A-fiber, *p* = 0.6919; C-fiber, *p* = 0.157; *n* = 15 animals; Fig. 4*C*).

These findings were then corroborated using soft, innocuous tactile stimulation (stroking the dorsal end of the forepaw with a cotton bud). Soft, innocuous cotton bud stimulation also elicited a robust increase in spike activity in both the DR (from baseline levels of 9.5 ± 2.1 Hz to 27 ± 5 Hz) and the SN (from baseline levels of 27.2 ± 4.4 Hz to 54.8 ± 4.3 Hz; *n* = 9 animals). With soft tactile stimulation in the presence of GABA, both the DR and SN firing frequency remained unchanged (DR, 29.6 ± 5.6 Hz; *p* > 0.999; SN, 59 ± 7.9 Hz; *p* > 0.999; one-way ANOVA; *n* = 9 animals; Fig. 4*D*,*E*). Spike sorting revealed that during soft tactile stimulation, there was no difference in the degree of filtering of those fibers identified as originating from either A- or C-fibers, and application of GABA during soft tactile stimulation did not significantly affect filtering (A-fiber, *p* = 0.265; C-fiber, *p* = 0.07; *n* = 9 animals; Fig. 4*F*).

Finally, proprioceptive stimulation elicited by moving the forearm vertically up and down, without tactile or noxious stimuli, allowed us to investigate the effects of GABA on spike activity through the DRG. Proprioceptive stimulation also elicited a robust increase in spike activity in both the DR (from baseline levels of 10.5 ± 1.6 Hz to 28 ± 4.3 Hz) and the SN (from baseline levels of 28.2 ± 4.3 Hz to 58.5 ± 8.4 Hz; *n* = 13 animals). In the presence of GABA, the DR and SN firing frequency remained unchanged (DR, 29.6 ± 5 Hz; *p* > 0.999; SN, 58.2 ± 10.1 Hz; *p* > 0.999; *n* = 13 animals; Fig. 4*G*,*H*). Spike sorting revealed no difference in filtering of those fibers identified as originating from A-fibers or from C-fibers during proprioceptive stimulation or proprioceptive stimulation in the presence of GABA (A-fiber, *p* = 0.937; C-fiber, *p* = 0.998; *n* = 13 animals; Fig. 4*I*).

### Endogenous GABA tone in the DRG contributes to the filtering of noxious, but not innocuous, activity

**Figure 5.**
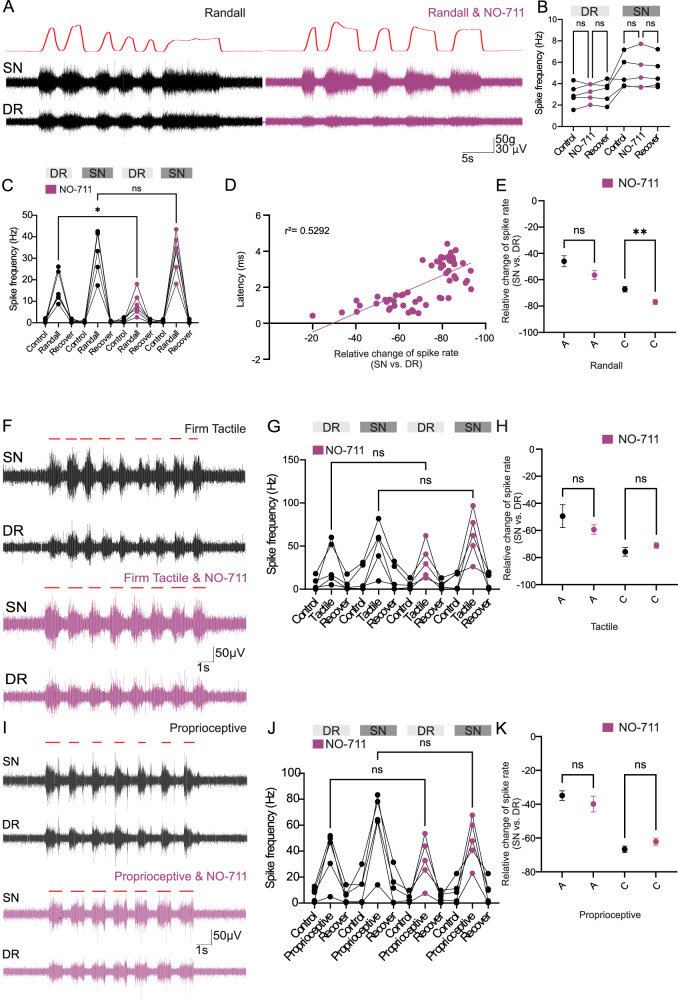
There is endogenous GABA tone at the DRG. ***A***, Example traces of SN and DR nerve activity during noxious Randall stimulation (top trace), in control conditions (black traces), and during infusion of the GAT-1 inhibitor, NO-711 (200 µM), directly into the DRG (purple traces). ***B***, Quantification of the effect of NO-711 on firing frequencies in the DR and SN under tonic/control conditions; ns denotes no significant difference (DR, *p* = 0.8751; one-way ANOVA; *n* = 5 animals and 15 stimulations; SN, *p* = 0.8751; one-way ANOVA; *n* = 5 animals and 15 stimulations). ***C***, Quantification of firing frequencies from the data exemplified in ***A***; * denotes *p* < 0.05; ns denotes no significant difference (DR, *p* = 0.0373; SN, *p* = 0.9996; one-way ANOVA). ***D***, Failure rate of identified spikes was correlated with the spike latencies (*R*^2^ = 0.3497; *Y* = −0.04049 * *X* − 0.9980; *p* < 0.0001). ***E***, Quantification of the relative change of the spike rate (sorted spikes) between SN and DR upon noxious Randall stimulation in control conditions (black) and under NO-711 (purple). “C-fibers” *p* = 0.0050; one-way ANOVA; *n* = 31; “A-fibers” *p* = 0.3211; *n* = 31 stimulations. * denotes *p* < 0.05; ns denotes no significant difference. ***F***, Example traces of nerve activity induced by firm, tactile (innocuous; black trace) in control conditions and during NO-711 infusion into the DRG (purple trace). Red lines denote stimulation period. ***G***, Quantification of firing frequencies from the data exemplified in ***G***; ns denotes no significant difference (DR, *p* = 0.9981; one-way ANOVA; *n* = 5 animals; SN, *p* = 0.1234; one-way ANOVA; *n* = 5 animals). ***H***, Quantification of the relative change of the spike rate (sorted spikes) between SN and DR upon hard tactile stimulation in control conditions (black) and under NO-711 (purple). “C-fibers” *p* = 0.7410; *n* = 14; “A-fibers” *p* = 0.4662; *n* = 9; ns denotes no significant difference. ***I***, Example traces of nerve activity induced by proprioceptive stimulation in the SN and DR under control conditions (black trace) and during NO-711 infusion into the DRG (purple trace). Red lines denote stimulation period. ***J***, Quantification of firing frequencies from the data exemplified in ***G***; ns denotes no significant difference (DR, *p* = 0.9986; SN, *p* = 0.8361; one-way ANOVA; *n* = 5 animals). ***K***, Quantification of the relative change of the spike rate (sorted spikes) between SN and DR upon proprioceptive stimulation in control conditions (black) and under NO-711 (purple). “C-fibers” *p* = 0. 9416; *n* = 16 stimulations; “A-fibers” *p* = 0.3710; *n* = 14 stimulations. ns denotes no significant difference. DR, dorsal root; SN, spinal nerve.

The GAT-1 GABA reuptake transporter inhibitor, NO-711, was injected directly into the DRG while performing dual DR–SN recordings using DAPP. This allowed us to confirm the presence of endogenous GABA tone in the DRG and determine if the effects of injecting exogenous GABA into the DRG can be mimicked by inhibiting uptake of endogenous GABA. NO-711 had no effects on spike activity during control conditions in either the DR or the SN. DR mean frequency was 8.9 ± 1.4 Hz in control conditions versus 9.5 ± 1.1 Hz with NO-711 (*n* = 5 animals and 15 trials; *p* = 0.875; one-way ANOVA). SN mean frequency was 16.11 ± 2.0 Hz in control conditions versus 15.26 ± 2.3 Hz with NO-711 (*n* = 5 animals; *p* = 0.999; one-way ANOVA; Fig. 5*A*,*B*). During noxious mechanical stimulation, spike frequency increased to 16.0 ± 2.9 Hz in the DR and to 33.82 ± 4.2 Hz in the SN (*n* = 6 animals and 26 stimulations). With NO-711 continually injected into the DRG during the noxious mechanical stimulation, this increase in spike activity was attenuated in the DR (to 8.74 ± 2.2 Hz; *p* = 0.0373) and remained unchanged in the SN (at 32.3 ± 3.7 Hz; *p* = 0.9996; one-way ANOVA; Fig. 5*C*). Spike sorting revealed that those fibers with the longest latencies, likely originating from C-fibers, were the most likely to be filtered (*R*^2^ = 0.5292; *Y* = −0.05242 * *X* − 1.537; *p* < 0.0001; Fig. 5*D*). There was no change in filtering in A-type fibers during noxious Randall stimuli [45.92 ± 4.2% reduction in spike rate (SN to DR)] in comparison with Randall stimuli with NO-711 (56.45 ± 3.5% reduction in the spike rate; *p* = 0.3211; *n* = 31 stimulations), but for C-type spikes, application of NO-711 significantly increased filtering of activity induced by noxious Randall stimuli (from 67.2 ± 2.1% reduction in the spike rate in Randall to 76.94 ± 2.0% reduction in the spike rate in Randall with NO-711; *n* = 44 stimulations; *p* = 0.0050; Fig. 5*E*). This suggests that, in accordance with earlier findings (Du et al., 2017), there is indeed endogenous GABA tone in the DRG, which selectively increases the probability of spike failure at the DRG in the unmyelinated fibers, reducing the frequency of nociceptive spikes being propagated to higher brain centers. Thus, increasing the levels of endogenous GABA in the DRG increases filtering of spike activity originating from unmyelinated fibers.

The effects of NO-711 upon firm, innocuous tactile stimulation (stroking the dorsal surface of the rat’s forepaw with the end of a plastic tweezer) were also examined. Upon firm, innocuous tactile stimulation, NO-711 was unable to attenuate the increased firing rates in both the SN and the DR (Fig. 5*F*–*H*). In the DR, spike frequency increased from 6.4 ± 3.3 Hz (control conditions) to 29.3 ± 11.1 Hz upon firm tactile stimulation and to 32.4 ± 8.9 Hz upon firm tactile stimulation in the presence of NO-711 (*n* = 5 animals; *p* = 0.998; one-way ANOVA). In the SN, spike frequency increased from 12.8 ± 6.9 Hz (control conditions) to 49.6 ± 12.2 and 62.6 ± 12 Hz upon firm tactile stimulation with and without NO-711, respectively (*n* = 5 animals; *p* = 0.123; Fig. 5*F*,*G*). There were no changes in the degree of filtering of spikes identified as originating from either A- (*p* = 0.447; *n* = 9) or C-type (*p* = 0.7410; *n* = 14; one-way ANOVA) fibers during tactile stimulation or with tactile stimulation in the presence of NO-711 (Fig. 5*H*). Proprioceptive stimulation resulted in an increase in spike frequency in both the SN and the DR; however, NO-711 again had no effects on the degree of such increase. In the DR, spike frequency upon proprioceptive stimulation increased from 6.8 ± 2.4 Hz (control conditions) to 36.8 ± 8.8 Hz and to 32.6 ± 7.9 Hz upon proprioceptive stimulation with or without NO-711, respectively (*p* = 0.9986; one-way ANOVA; *n* = 5 animals). In the SN, spike frequency increased from 12.5 ± 5.4 Hz (control conditions) to 60.5 ± 12.3 Hz upon proprioceptive stimulation. This increase in spike frequency at the SN was not significantly affected by NO-711 (47.9 ± 7.8 Hz; *p* = 0.836; *n* = 5 animals; Fig. 5*I*,*J*). There were no changes in the degree of filtering of spikes identified as originating from either A- (*p* = 0.3710; *n* = 5 animals) or C-type (*p* = 0.9416; *n* = 5 animals; one-way ANOVA) fibers during proprioceptive stimulation or with proprioceptive stimulation in the presence of NO-711 (Fig. 5*K*).

## Discussion

A growing body of literature suggests that axonal bifurcations of sensory neurons within DRG (and trigeminal ganglia) function as a “gate” or filter for transmission of somatosensory information from the peripheral nerves to the CNS (Gemes et al., 2013; Sundt et al., 2015; Du et al., 2017; Kent et al., 2018; Al-Basha and Prescott, 2019; Chao et al., 2020; Fuller et al., 2023). One mechanism for such filtering is the intrinsic GABAergic system (Hayasaki et al., 2006; Hanack et al., 2015; Du et al., 2017; Hao et al., 2023). Indeed, DRG and TG neurons abundantly express GABA_A_ receptors (reviewed here; Wilke et al., 2020), and some of them can produce and release GABA in an activity-dependent manner (Hayasaki et al., 2006; Hanack et al., 2015; Du et al., 2017; Hao et al., 2023). Additionally, satellite glia releases endogenous endozepine peptide, DBI, which activates and potentiates GABA_A_ channels in the DRG (Li et al., 2024). Consequently, direct pharmacological activation of GABA_A_ channels alleviates acute and chronic pain in vivo (Du et al., 2017; Hao et al., 2023; Li et al., 2024). Despite this progress, direct evidence for such gating/filtering is hard to obtain due to the anatomical difficulties in accessing the ganglia and different aspects of the corresponding nerves in vivo. Several in vivo electrophysiological recording approaches testing the idea of spike filtering at the DRG have been developed (Al-Basha and Prescott, 2019; Chao et al., 2020; Hao et al., 2023), and these were indeed instrumental in establishing the filtering phenomenon. Yet, being in vivo recordings, these require general anesthesia, which is not ideal, as anesthetics are likely to interfere with nociception and the GABAergic system specifically (Hemmings, 2009).

Here we modified the WHBP preparation (Paton, 1996) to investigate peripheral somatosensory system. This decerebrate preparation provides unparalleled access to peripheral nerves, the DRG, and spinal pathways for recording, stimulation (electrical and sensory), and direct drug application; importantly, it does not require ongoing anesthesia. The DAPP was specifically designed to examine the propagation of peripheral nerve spikes from the SN (pre-DRG) to the DR (post-DRG), allowing real-time comparison of electrical activity; however, it can be used for multiple other research applications. The preparation is stable for several hours and allows direct access to multiple sites within the somatosensory system for stable, repeatable, and reproducible recordings. In combination with digitized sensory stimulation (e.g., digital Randall–Selitto) and spike sorting/matching algorithms, DAPP allows for precise quantification of sensory input/nerve activity output, including the ability to quantify signal modulation and processing within peripheral and spinal somatosensory pathways. Conduction velocities observed in the DAPP preparation were consistent with the range of published values for A- and C-fiber types (Gemes et al., 2013; Hao et al., 2023). Nonetheless, minor deviations may occur due to experimental conditions such as perfusate temperature.

Using DAPP, we demonstrate that both exogenous and endogenous GABA strongly inhibits nociceptive input at the DRG by increasing spike filtering and reducing nociceptive afferent traffic from the periphery to the CNS, presumably due to T-junctional failure (Du et al., 2017; Hao et al., 2023). Thus, the application of both GABA (Fig. 3) and the GABA reuptake inhibitor, NO-711 (Fig. 5), to the DRG enhanced filtering of spikes induced by noxious mechanical stimulation from the SN to the DR. Consistent with previous observations (Hao et al., 2023), activity induced by innocuous mechanical or proprioceptive stimulation was not affected by GABA nor NO-711 (Figs. 4 and 5). Also, tonic activity was unaffected by neither GABA nor NO-711. The efficacy of NO-711 to induce DRG filtering of noxious activity indicates that there is endogenous, inhibitory, and activity-dependent GABA release at the DRG, which can be maximized by inhibition of GABA reuptake; these findings are consistent with earlier reports (Du et al., 2017 ; Hao et al., 2023 ). On the other hand, the lack of GABA inhibition of tonic filtering suggests that spontaneous activity is largely mediated by fibers that are less sensitive to GABA-dependent filtering and that other mechanisms controlling DRG filtering must exist.

Our spike sorting/matching method (Hao et al., 2023) allowed us to identify and cluster spikes and allowed those spike units that were most likely to fail to be identified. This method revealed that among the spikes induced by noxious mechanical stimulation, the longest latency units (likely the C-fibers) are those that are the most likely to fail upon GABA or NO-711 application. A selective increase of filtering in C-fibers was also reported for electric field stimulation (neuromodulation) of the DRG (Chao et al., 2020); hence, this may be a common feature for some T-junction filtering mechanisms. One hypothesis for such preferential filtering in the C-fibers is that, because unmyelinated C-fibers have much shorter stem axons compared with myelinated fibers (Hao et al., 2023), the electrotonic coupling between the soma and the T-junction in C-fibers is much higher compared with A-fibers, allowing for somatic ion channels, such as GABA_A_, to have a higher influence over the spikes propagating through the T-junction (Hao et al., 2023). This, however, requires further investigation, and other mechanisms could be at play.

The DRG is an exciting therapeutic target for pain treatment, without the added complications of CNS-associated side effects. Targeting DRG allows for modulation of nociception prior to entry of the CNS, an approach already being used clinically in DRG neuromodulation (Deer et al., 2013; Krames, 2015; Esposito et al., 2019; Eldabe et al., 2022). There are two further features of interest: (1) nociceptive C-fibers are more susceptible to DRG filtering, as compared with A-fibers (this study; Chao et al., 2020; Hao et al., 2023), and (2) blood–DRG barrier is much more open to circulation, as compared with hematoencephalic barriers of other segments of the nervous system (Lund et al., 2024). These features may open an opportunity to develop drugs, such as GABA-mimetics or allosteric modulators with reduced blood–brain barrier permeability. Such drugs may still penetrate the DRG and reduce nociception without affecting other haptic sensations mediated by A-fibers and without CNS side effects. Understanding this selective filtering provides mechanistic insight relevant to refining DRG neuromodulation for patient-specific pain treatment.

### Technical challenges

To achieve adequate oxygenation via arterial perfusion in DAPP, it is necessary to use high perfusion rates of up to 30 ml/min (adjusted for each preparation based on respiratory rhythm). Despite this, appropriate lower-body circulation is still compromised in this preparation. Hence, recordings were obtained from upper limbs (the median nerve) and not nerves innervating the lower limbs (e.g., sciatic nerve). Although the recorded pathways (C8 DRG and median nerve) remain anatomically intact in this preparation, we cannot exclude the possibility that systemic trauma associated with the broader surgical exposure may induce a systemic nociceptive tone, which could influence DRG neuron excitability and function. The spontaneous activity of afferent fibers was reported in several types of in vivo preparations (Wall, 1960; Jänig et al., 1968), as well as in our recent study on anesthetized rats (Hao et al., 2023). The ongoing proprioceptive input from muscle spindle afferents may contribute to such activity. While spontaneous firing of C-type fibers is less common under normal physiological conditions, it has been reported under sensitized, injured, or inflamed states (Ali et al., 1999; Wu et al., 2001; Serra et al., 2012). Given the systemic surgical exposure in our preparation, it is plausible that spontaneous activity in C-fibers could reflect a mild neurogenic activation or early injury-related sensitization, particularly in mechanoinsensitive or peptidergic subpopulations. Additionally, the high perfusion rates may affect the thermal sensitivity of the skin, due to higher than physiological thermal convection rates. Thus, while responses to mechanical stimulation reported here were always robust and reproducible, a much higher variability in response to thermal stimulation (both heat and cold) was observed; we therefore did not include these data.

## Conclusions

This is the first study, to our knowledge, to show that there is both tonic and activity-dependent spike filtering at the DRG in unanesthetized rats. Using the DAPP, we have shown that exogenous and endogenous GABAergic signaling at the sensory neurons of the DRG are capable of selectively filtering noxious stimuli from reaching the spinal cord. This may open new avenues for the long-term management of chronic pain. Additionally, we describe an approach for mechanistic evaluation of input–output relationships of the intact somatosensory system in the in vivo-like preparation devoid of general anesthesia.

## Data Availability

The code for spike sorting analysis is available at GitHub (https://github.com/pnm4sfix/SpikePropagation).
